# P142: Education of key personnel in infection control in hospitals in the capital region of Copenhagen

**DOI:** 10.1186/2047-2994-2-S1-P142

**Published:** 2013-06-20

**Authors:** AF Madsen, D Mogensen, AJ Jørgensen, H Neustrup

**Affiliations:** 1 Dept.of Clinical Microbiology, University of Copenhagen, Hvidovre Hospital, DK-2650 Hvidovre, Denmark; 2 Dept.of Clinical Microbiology, Herlev Hospital, DK-2730 Herlev, Denmark; 3 Dept.of Clinical Microbiology, Hilleroed Hospital, DK-3400 Hilleroed, Denmark; 4 Dept. of infection control 9101, Rigshospitalet, DK-2100 Copenhagen, Denmark

## Introduction

According to the infection control programme 2011-2012, a need for education of various professionals in infection prevention and control is described. The Capital Regions quality-council has aimed to reduce Hospital acquired infections by 50% over a 3-year period.

## Methods

The education is primarily intended for health care workers with a bachelor degree (RN, MD). The education extends for 5 days and is a combination of theoretical training, case studies in groups, and in presentation of the studies. The education is offered 3 times a year with 25 students per course.

The focus is the organization of the infection prevention and control nationally and locally, helping to create a link between overall policy objectives and infection prevention and control at each department / hospital.

The educational programme aims to ensure:

- A uniform basis for practicing infection control for the front line personnel

- Networking across hospitals

- Implementation of accreditation standards and guidelines in infection control and patient safety in cooperation with managers and employees

- Introduction to international, regional and local infection control guidelines

- Evaluation by a questionnaire

## Results

The participants (N=159) evaluated the education by a questionnaire. There are 115-answered questionnaires, a response rate of 72% (N=115). The form is mainly constructed with Likerts Scale, where a scoring on 4 means very good and 0 means very bad. Results are above “good” and does not fluctuating over time (2011-2012).

- Academic achievement 3.22

- Clinically usefull 3.10

- Professional level 3.18

## Conclusion

According to the questionnaire survey 115/159 (72%) were satisfied with the contents and education.

After education of 159 key infection control personnel, local initiatives have been reinforced at the hospitals within the infection-control area. The initiative is followed up by 2-4 annual theme days with current topics to strengthen the networking.

The education is adapted to the Capital Region’s overall strategy on infection control.

## Disclosure of interest

None declared

